# Brain glucose metabolism on [18F]-FDG PET/CT: a dynamic biomarker predicting depression and anxiety in cancer patients

**DOI:** 10.3389/fonc.2023.1098943

**Published:** 2023-05-25

**Authors:** Xue Yang, Guangxia Yang, Ruojun Wang, Yanjuan Wang, Shengyi Zhang, Jian Wang, Chunjing Yu, Zeqin Ren

**Affiliations:** ^1^ Department of Neurology, Affiliated Hospital of Jiangnan University, Jiangnan University, Wuxi, Jiangsu, China; ^2^ Department of Rheumatology, Affiliated Hospital of Jiangnan University, Jiangnan University, Wuxi, Jiangsu, China; ^3^ Department of Nuclear Medicine, Affiliated Hospital of Jiangnan University, Jiangnan University, Wuxi, Jiangsu, China; ^4^ Department of Orthopaedics, The Ninth People’s Hospital of Wuxi, Affiliated to Suzhou University, Wuxi, Jiangsu, China; ^5^ Department of Rehabilitation, The First Affiliated Hospital of Dali University, Dali, Yunnan, China

**Keywords:** glucose metabolism, cancer, depression, anxiety disorder, positron emission

## Abstract

**Objectives:**

To explore the correlation between the incidence rates of depression and anxiety and cerebral glucose metabolism in cancer patients.

**Methods:**

The experiment subjects consisted of patients with lung cancer, head and neck tumor, stomach cancer, intestinal cancer, breast cancer and healthy individuals. A total of 240 tumor patients and 39 healthy individuals were included. All subjects were evaluated by the Hamilton depression scale (HAMD) and Manifest anxiety scale (MAS), and were examined by whole body Positron Emission Tomography/Computed Tomography (PET/CT) with 18F-fluorodeoxyglucose (FDG). Demographic, baseline clinical characteristics, brain glucose metabolic changes, emotional disorder scores and their relations were statistically analyzed.

**Results:**

The incidence rates of depression and anxiety in patients with lung cancer were higher than those in patients with other tumors, and Standard uptake values (SUVs) and metabolic volume in bilateral frontal lobe, bilateral temporal lobe, bilateral caudate nucleus, bilateral hippocampus, left cingulate gyrus were lower than those in patients with other tumors. We also found that poor pathological differentiation, and advanced TNM stage independently associated with depression and anxiety risk. SUVs in the bilateral frontal lobe, bilateral temporal lobe, bilateral caudate nucleus, bilateral hippocampus, left cingulate gyrus were negatively correlated with HAMD and MAS scores.

**Conclusion:**

This study revealed the correlation between brain glucose metabolism and emotional disorders in cancer patients. The changes in brain glucose metabolism were expected to play a major role in emotional disorders in cancer patients as psychobiological markers. These findings indicated that functional imaging can be applied for psychological assessment of cancer patients as an innovative method.

## 1 Introduction

Malignant tumor is a disease that seriously threatens human health. It is estimated that the global cancer burden will reach 28.4 million cases by 2040, an increase of 47% over 2020 (1). Malignant tumor not only causes patients physical pain, but also leads to varying degrees of mental stress. With the progress of medical technology and the change of therapeutic model, malignant tumor has become a somatopsychic disease. Since most tumors cannot be completely cured, tumor patients are often accompanied by obvious psychological changes, the most common symptoms of which are anxiety and depression. Tumor-related anxiety and depression refers to the pathological mental disorders or syndrome during the diagnosis and treatment of malignant tumors. Symptoms mainly include decreased interest, lack of energy, lack of physical strength, pessimism, self-incrimination and suicide tendency. Previous studies have shown that the in cadence of depression in tumor patients is 1.5%~50.0% (2).It should be emphasized that tumor-related anxiety and depression is a group of symptoms or states caused by tumor based diseases, not psychotic anxiety and depression. Anxiety and depression significantly increase cancer patients’ risk of noncompliance with treatment, and contribute to a poorer treatment response and increased rates of hospital admission, worst of all, it can lead to mortality and even suicide (3–7). Therefore, early recognition of depression and anxiety is crucial to the prognosis of cancer patients.

Positron emission tomography/computed tomography (PET/CT) with 18F-fluorodeoxyglucose (FDG) has been extensively used in oncology for providing metabolic information and studying cancer staging, therapeutic response etc (8, 9). In recent years, resting-state PET/CT techniques can also be applied to validate hypotheses concerning the changes in functional connectivity that occur in various kinds of diseases such as schizophrenia, Alzheimer’s disease (10), depression (11), diabetic patients (12) and normal aging (13). 18F-FDG uptake was measured with PET technology to assess the cerebral glucose metabolic rate at rest, as a representative of neuronal activity (14). Numerous studies have demonstrated that the brain glucose metabolism changes in patients with depression before and after antidepressant treatment. Previous studies have shown that local glucose metabolism increases in the left dorsal prefrontal cortex and anterior cingulate cortex were observed by 18F-PET/CT in patients with refractory depression after treatment, which is highly correlated with the improvement of depression (15). In the past, abnormal glucose metabolism in the brain of patients with various malignant tumors has been detected by 18F-PET/CT (16, 17), which suggests that there may be tumor-related depression. PET/CT is commonly used in tumor diagnosis, and tumor patients are often accompanied by emotional abnormalities. Therefore, PET/CT evaluation of local brain glucose metabolism changes in tumor patients is helpful for early detection and intervention of tumor-related depression.

The purpose of this study was to compare the correlation between the incidence rate of depression and anxiety and cerebral glucose metabolism in different tumors, distinct clinical stages and pathological types. This study revealed the characteristics of brain glucose metabolism changes in cancer patients and their correlation with emotional disorders. Brain glucose metabolic changes can hopefully be regarded as a clinical neurobiological label to evaluate emotional disorder for cancer patients.

## 2 Methods

Patient recruitment. A total of 240 tumors patients and 39 matched controls were recruited from the Affiliated Hospital of Jiangnan University, from January 2022 to September 2022.

### 2.1 Tumor group

Inclusion criteria were as follows: (I) The participants were not aware of the disease, aged 18-80 years old, regardless of gender. (II)A clinical suspicion of malignancy. (III) No previous organic diseases such as heart, lung, liver, pancreas, kidney diseases, no metabolic diseases of hyperthyroidism, and no history or family history of neuropsychiatric diseases. (IV) No history of cerebrovascular accident, epilepsy, brain trauma or brain surgery. (V) The patient had no conscious disorder and severe cognitive disorder, and can complete the Hamilton depression scale (HAMD) and manifest anxiety scale (MAS). Exclusion criteria were as follows: (I) Failure to successfully complete PET/CT examination or emotional scale evaluation. (II) Later, brain PET images cannot meet the research needs. (III) Confirmed or suspected meningeal or brain metastasis. (IV) No pathological evidence of tumor was obtained. (V) Parallel participants in other clinical research.

### 2.2 The control group

Those who planned to perform 18F-PET/CT examination in the Affiliated Hospital of Jiangnan University and who needed tumor screening for various reasons. Inclusion criteria were as same as tumor group (I) ~(V). Exclusion criteria were as same as tumor group (I) ~(V). The study protocol was approved by the Ethics Committee of the Affiliated Hospital of Jiangnan University, and all patients or their relatives gave written informed consent.

### 2.3 Evaluation of emotional disorders

Hamilton Depression Scale (HAMD) and Dominant Anxiety Scale (MAS) were used to evaluate the patients who were clinically suspected of malignant tumors and who were to be examined with 18F-FDG PET/CT in the whole body (brain + trunk).All subjects received medical history collection, physical examination, and HAMD and MAS evaluation within one week after the diagnosis of malignant tumor. Depression was defined as HAMD score ≥ 8, and anxiety was defined as MAS score ≥ 14. The severity ranges for the HAMD: no depression (0-7); mild depression (8-16); moderate depression (17-23); and severe depression (≥24). The severity ranges for the MAS: no anxiety (0-14); mild anxiety(15-39); and severe anxiety (≥40) (18).

### 2.4 PET image acquisition

All patients were obliged to fast for at least 6 h before 18F-FDG (5.55 MBq/kg) intravenous injection. Acquisition was conducted at 60 min after the injection. The patient was positioned in a supine position on the scanner bed. Imaging data were purchased from the skull to the thigh with a 1.5 min/bed position. PET/CT scanner from Siemens (Biograph True Point PET/CT) collects a series of static images of the trunk (from skull to thigh), and uses CT data to conduct attenuation correction and positioning of PET images. PET/CT served to differentiate the tumor site, abnormal metabolism and metastatic lymph nodes. PET/CT TNM staging report was made by two experienced nuclear physicians based on the patient’s PET/CT examination results to analyze the patient’s tumor TNM staging. Pathological TNM (pTNM) represents the pathological classification of a tumor. Both clinical and pathological stage involve describing the extent of the primary tumour (T category), involvement of regional lymph nodes (N category), and spread (or metastasis) to distant sites (M category) at the different time- points described above. The T, N, and M categories can then be combined into Stage groups I, II, III, and IV (19). Typically, stage I tumours are confined to the organ of origin, and stage IV tumours have distant metastatic disease. American Joint Committee on Cancer (AJCC) eighth edition staging manual was used for T, N, and M staging (20).

### 2.5 Data processing

In this study, 18F-FDG uptake was measured using PET technology to assess the resting-state cerebral glucose metabolism rates as a proxy for neuronal activity. All imaging data were processed using the PMOD PNEURO software tool (version 4.0 PMOD Technologies Ltd, Zu rich, Switzerland).

On the PMOD software, position correction and normalization are performed on the image to make the PET image correspond to the spatial coordinates of the brain atlas provided by the software. The brain regions were divided into 116 brain parts (58 regions in each hemisphere) through stereoscopic analysis, and the standard uptake value (SUV) and metabolic volume of each brain region was calculated quantitatively, corresponding approximately to automated anatomical labeling (AAL-VOIs) atlas, to assess the metabolism of the study area. Draw the ROI and extract the average SUV value and Metabolic volume of the ROI. Finally, the image was statistically processed.

Metabolic volume is defined as the sum of the volume of voxels with SUV surpassing a threshold value in a volume-of-interest (VOI). The highest voxel value on 18F-FDG PET/CT was determined as SUVmax. The volume of the brain regions with SUV ≥ 2.5 was determined as Metabolic volume (ml) (21).

Z value was introduced into the software, which can be used to compare SUV values of normal people and test patients. The z-score defines the deviation of a sample with respect to the mean of a distribution. It is defined by the formula z=(x-m)/σ

Where x is the sample value, m the sample mean, and σ the standard deviation of the distribution. Therefore, z describes in the deviation from the mean in number of standard deviations and is positive, when the sample is above mean, and negative when below. M is Software Average SUV value.

### 2.6 Statistical analysis

SAS 9.3 statistical software was used for data analysis. The measurement data were analysed by ANOVA, expressed as “*x ± s*”. The counting data were compared by the *x^2^* test. *P* value <0.05 was considered significant. Prism statistical software was used for the HAMD depression scale score, MAS anxiety scale score, SUV value and metabolic volume among different tumor and lung cancer stages and pathological grades were analyzed by variance. *P* value <0.05 was considered significant. Pearson correlation analysis was conducted on the average SUV and HAMD depression scale score and MAS anxiety scale score in the abnormal activation area, with r>0.5 or r<- 0.5 as the correlation.

## 3 Results

### 3.1 Demographic and baseline clinical characteristics

The clinical data collected after recruitment include: sociodemographic characteristics: age, gender, marital status, place of residence, employment status before surgery and education level, medical history and complications: smoking, drinking, hypertension, hyperlipidemia, coronary heart disease, atrial fibrillation, diabetes, blood glucose level, BMI value, Tumor characteristics: tumor pathological grade, TNM stage, and degree of clinical treatment.

A total of 240 cancer patients and 39 matched controls were included in the analysis. There were 240 adult patients with newly diagnosed and confirmed cancer, including those with breast (12.5% of cancer patients), lung (41.25%), intestinal (17.92%), stomach (12.5%), and head and neck tumor (15.83%) (**Table 1**).

**Table 1:** Baseline and demographic clinical characteristics.

	Ctrl group 39	Lung cancer 99	Head and neck tumor38	Gastric cancer 30	Intestinal cancer43	Breast cancer30	P value
Sex							<.0001
male	19	67	27	18	25	0	
female	20	32	11	12	28	30	
Age	57.97± 7.75	62.19± 8.78	58.84± 9.46	60.23± 9.08	62.44± 8.14	60.20± 8.45	0.0655
Smoke(yes/no)	7/32	25/74	8/30	5/25	4/39	0/30	0.0253
Drink(yes/no)	5/34	16/83	8/30	5/25	5/38	0/30	0.2013
Hypertension(yes/no)	8/31	27/72	9/29	12/18	16/27	5/25	0.1947
Diabetes(yes/no)	2/37	10/89	4/34	3/27	3/40	0/30	0.4868
FBG(mmol/l)	5.99 ± 0.99	5.98 ± 1.55	5.87 ± 0.92	5.76 ± 0.90	6.04 ± 0.92	6.17 ± 1.17	0.8327
Hyperlipidemia (yes/no)	2/37	3/96	1/37	1/29	2/41	0/30	0.9145
Coronary heart disease(yes/no)	6/33	10/89	2/36	5/25	10/33	2/28	0.1434
Atrial fibrillation(yes/no)	1/38	1/98	0/38	0/30	0/43	0/30	0.7651
BMI(kg/m2)	22.07 ± 2.13	22.72 ± 2.72	22.86 ± 2.39	21.73 ± 2.61	22.86 ± 1.50	21.67 ± 1.92	0.0538
Level of education							0.0532
Junior high school or less	15	65	19	19	22	14	
High school	15	22	13	8	20	11	
Undergraduate Graduate or above	9	12	6	3	1	5	
Marry status							0.977
Single	0	3	0	0	0	0	
Divorced	3	5	2	2	4	2	
Widowed	3	3	2	1	3	2	
Married	33	91	34	27	36	36	
Place of abode							0.3125
City	23	63	26	15	20	14	
Cities and towns	10	26	8	12	20	11	
Country	6	10	4	3	3	5	
Working status							0.6310
working	27	69	26	21	25	17	
non-working	12	30	12	9	18	13	

Age, marital status, place of residence, employment status and education level before operation, alcohol consumption, hypertension, hyperlipidemia, coronary heart disease, atrial fibrillation, diabetes, blood glucose level, BMI value, tumor pathological classification, TNM stage were not statistically significant among groups. Gender was statistically significant because all breast cancer patients were female. The smoking rate of lung cancer patients has statistical difference. Due to different tumor types, clinical stages and pathological grades, treatment plans are also various, leading to statistical significance between surgical treatment, chemotherapy and targeted treatment (**Tables 1**, **2**).

**Table 2:** Tumor conditions and treatment.

	Lung cancer	Head and neck tumor	Gastric cancer	Intestinal cancer	Breast cancer	p
Pathological grade						0.8531
G1	31	11	10	12	10	
G2	25	11	5	7	7	
G3	43	16	15	24	13	
TNM staging of patients						0.0755
I	29	6	9	11	11	
II	22	18	1	8	11	
II	11	3	6	8	6	
IV	37	11	14	16	2	
Treatment mode of patients(yes/no)	94/5	34/4	29/1	43/0	30/0	0.1339
Surgical treatment (yes/no)	54/45	19/19	28/2	35/8	28/2	<.0001
Radiotherapy (yes/no)	27/72	6/32	0/30	0/43	10/20	<.0001
Chemotherapy(yes/no)	52/47	20/18	13/17	26/17	18/12	0.6198
Targeted therapy(yes/no)	39/60	10/28	7/23	18/25	0/30	0.0004

### 3.2 Incidence of depression and anxiety and SUVs of different brain regions in patients with different tumors

**Figure 1:**
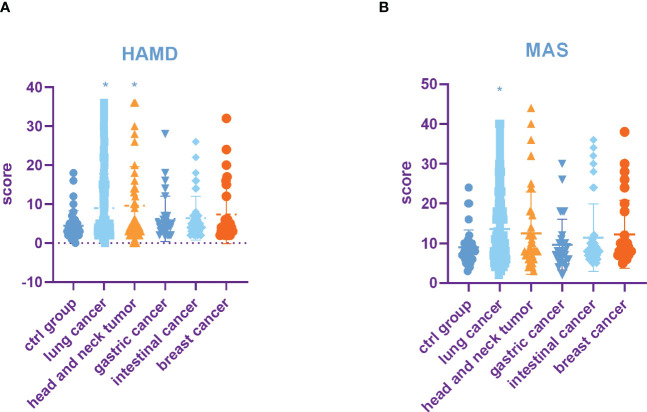
Incidence rate of depression and anxiety among tumors in different parts. **(A)** HAMD score among different tumors. **(B)** MAS score among different tumors. All data represent mean ± SD, *P<0.05.

2.1 Incidence rates of depression and anxiety in different types of tumors:

HAMD and MAS were used to assess the incidence of depression and anxiety. The HAMD score in the lung cancer group and head and neck tumor group was significantly higher than that in control group, stomach cancer, intestinal cancer, and breast cancer. Moreover, compared with the control group, head and neck tumor group, stomach cancer, intestinal cancer, and breast cancer, the MAS score in the lung cancer group increased significantly (**Table 3**, **Figures 1A, B**).

**Table 3:** Incidence rates of depression and anxiety in different tumors.

	Ctrl group 39	Lung cancer 99	Head and neck tumor38	Gastric cancer 30	Intestinal cancer43	Breast cancer30
HAMD	5/34(12.8%)	36/63(36.4%)	13/25(34.2%)	5/25(16.7%)	7/36(16.3%)	7/23(23.3%)
MAS	5/34(12.8%)	31/68(31.3%)	10/28(26.3%)	5/25(16.7%)	7/36(16.3%)	7/23(23.3%)

**Figure 2:**
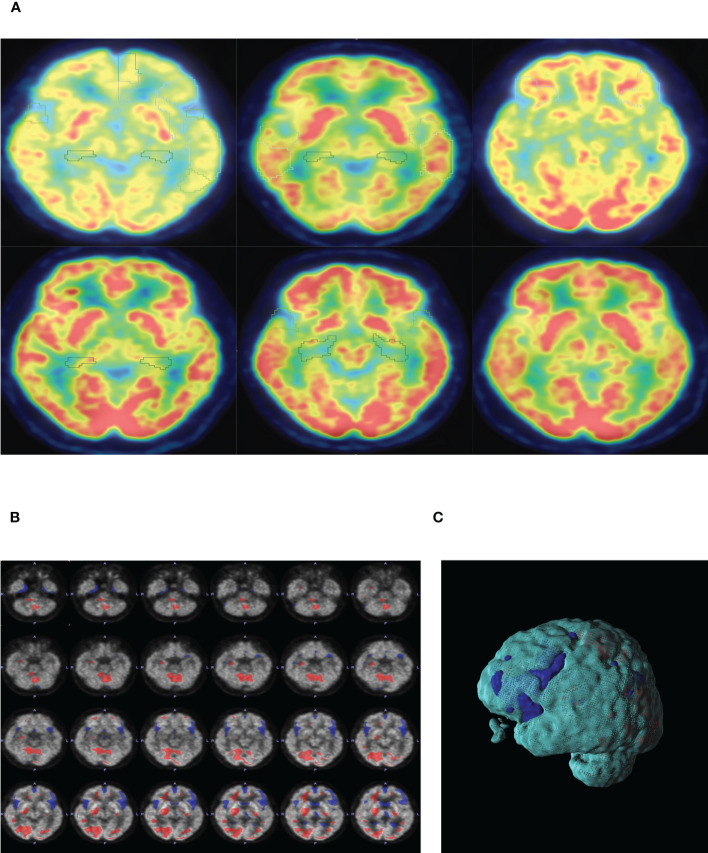
**(A)** PET-CT representative diagram of the brain in patients with different tumors. From left to right: lung cancer, head and neck tumor, gastric cancer, intestinal cancer, breast cancer and ctrl group. Low metabolism is framed in different brain regions (SUV<6). **(B)** Representative diagram of lung cancer patients. SUVs of lung cancer patients were analyzed by POMD. Compared with the standard value in POMD software, blue represents a decrease in SUVs and red represents an increase in SUVs(Z=3). **(C)** 3D reconstruction of B.

2.2 SUVs and metabolic volume of different brain regions in patients with different tumors:

A comparison of whole-brain glucose uptake was performed to detect neuronal activity at the regional level. SUVs of each brain region were used to evaluate glucose metabolism. It was found that the SUVs of the bilateral frontal lobes, bilateral temporal lobes, bilateral caudate nuclei, bilateral hippocampus, and left cingulate gyrus in the lung cancer group were significantly lower than those in the control group. Compared with the control group, SUVs of the bilateral frontal lobes, bilateral temporal lobes, bilateral hippocampus and left cingulate gyrus in head and neck tumors significantly decreased. Moreover, the SUVs of the bilateral occipital lobe in the lung cancer, intestinal cancer and breast cancer group were significantly higher than those in the control group. The metabolic volume of the bilateral frontal lobes, bilateral temporal lobes, left caudate nuclei, bilateral hippocampus, and left cingulate gyrus in the lung cancer group and head and neck tumors were significantly lower than those in the control group (**Tables 4**, **5**, **Figures 2A–C**).

**Table 4:** SUV values of different brain regions in patients with different tumors.

	Ctrl group	Lung cancer	p	Head and neck tumor	p	Gastric cancer	p	Intestinal cancer	p	Breast cancer	p
L-frontal	7.196 ± 1.007	6.284 ± 1.220	0.0002	6.335 ± 1.021	0.0052	6.661 ± 0.895	0.1987	6.996 ± 1.114	0.8909	7.079 ± 1.431	0.9919
R-frontal	7.226 ± 0.873	6.414 ± 1.231	0.0006	6.567 ± 1.052	0.0378	6.878 ± 0.764	0.5486	6.975 ± 1.080	0.7429	7.136 ± 1.263	0.9966
L-temporal	7.425 ± 0.951	6.411 ± 1.257	<0.0001	6.344 ± 1.359	0.0007	7.251 ± 1.006	0.9652	7.467 ± 1.252	0.9997	7.509 ± 1.414	0.9984
R-temporal	7.605 ± 0.888	6.704 ± 1.258	0.0003	6.657 ± 1.175	0.0020	7.429 ± 1.054	0.9550	7.507 ± 1.155	>0.9947	7.637 ± 1.247	0.9999
L-caudate	7.883 ± 0.736	7.341 ± 1.150	0.0215	7.391 ± 1.041	0.1267	7.807 ± 0.898	0.9980	7.859 ± 0.995	0.9999	7.718 ± 0.922	0.9399
R-caudate	7.963 ± 0.702	7.489 ± 1.055	0.0446	7.532 ± 1.048	0.1926	7.834 ± 0.864	0.9744	7.799 ± 1.036	0.9503	7.775 ± 0.926	0.8888
L-hippocampus	7.693 ± 0.847	6.507 ± 0.882	<0.0001	6.819 ± 1.163	0.0001	7.530 ± 0.717	0.9113	7.655 ± 0.774	0.9997	8.079 ± 0.999	0.2688
R- hippocampus	7.719 ± 0.821	6.601 ± 0.901	<0.0001	6.810 ± 1.108	<0.0001	7.468 ± 0.692	0.6504	7.650 ± 0.767	0.9962	8.104 ± 0.952	0.2574
L-cingulum	8.355 ± 0.627	7.920 ± 0.713	0.0126	7.861 ± 0.911	0.0212	8.248 ± 0.591	0.9674	8.333 ± 0.818	0.9998	8.189 ± 0.937	0.8343
L-occipital	8.097 ± 0.579	8.666 ± 0.823	0.0002	8.304 ± 0.740	0.5737	8.382 ± 0.459	0.3288	8.672 ± 0.589	0.0015	8.660 ± 0.799	0.0059
R- occipital	8.115 ± 0.496	8.609 ± 0.874	0.0026	8.267 ± 0.785	0.8378	8.318 ± 0.459	0.6858	8.726 ± 0.627	0.0013	8.636 ± 0.901	0.0194

**Table 5:** Metabolic volume of different brain regions in patients with different tumors.

	Ctrl group	Lung cancer	p	Head and neck tumor	p	Gastric cancer	p	Intestinal cancer	p	Breast cancer	p
L-frontal	1.99 ± 1.5182	11.21 ± 1.980	<0.0001	11.59 ± 1.783	0.0162	12.42 ± 1.433	0.8196	12.62 ± 1.951	0.9356	12.61 ± 2.425	0.9648
R-frontal	12.69 ± 2.334	11.35 ± 2.215	0.061	11.20 ± 1.571	0.0159	12.58 ± 1.360	>0.9999	12.68 ± 1.743	>0.999	12.70 ± 2.192	>0.999
L-temporal	15.58 ± 1.262	13.80 ± 1.897	<0.0001	14.07 ± 1.173	0.0011	15.29 ± 1.333	0.9781	15.38 ± 1.617	0.9938	15.53 ± 1.512	>0.9999
R-temporal	15.74 ± 1.131	14.23 ± 1.966	<0.0001	14.61 ± 1.508	0.0295	15.69 ± 1.451	>0.9999	15.58 ± 1.436	0.9973	15.81 ± 1.440	>0.9999
L-caudate	9.734 ± 0.7927	9.081 ± 1.129	0.0305	8.797 ± 1.116	0.0046	9.590 ± 0.8984	0.9953	9.885 ± 1.338	0.9907	9.667 ± 1.413	0.9999
R-caudate	9.794 ± 0.7956	9.314 ± 0.9651	0.1669	9.129 ± 0.9542	0.0718	9.599 ± 0.9133	0.9750	9.841 ± 1.388	>0.9999	9.698 ± 1.392	0.9991
L-hippocampus	9.649 ± 1.050	8.574 ± 0.9141	<0.01	8.829 ± 1.133	0.0059	9.51 ± 0.8660	0.9933	9.586 ± 0.8301	0.9998	10.15 ± 1.414	0.3178
R- hippocampus	9.713 ± 1.048	8.652 ± 0.9159	<0.0001	8.814 ± 1.092	0.0006	9.514 ± 0.8891	0.9557	9.9607 ± 0.8341	0.9959	10.17 ± 0.9507	0.3351
L-cingulum	11.58 ± 0.8462	10.99 ± 0.9740	0.0298	10.77 ± 1.219	0.0073	11.57 ± 1.133	>0.9999	11.36 ± 1.019	0.9196	11.20 ± 1.041	0.6490

### 3.3 Incidence rates of depression and anxiety and SUVs and metabolic volume of different brain regions in lung cancer patients with different TNM stages and pathological grades

The incidence rates of depression and anxiety in lung cancer patients were higher than that in other tumors, and the SUVs and metabolic volume in several brain regions of lung cancer were lower. Therefore, we further analyzed the incidence of depression and anxiety in lung cancer patients with different TNM stages and different pathological grades.

#### 3.3.1 Incidence rates of depression and anxiety and SUVs and metabolic volume of different brain regions in lung cancer patients with different TNM stages

**Figure 3:**
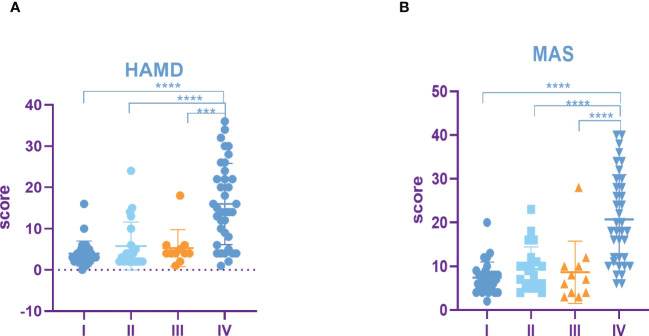
Incidence rate of depression and anxiety among lung cancer in different TNM periods. **(A)** HAMD score among different TNM periods of lung cancer. **(B)** MAS score among different TNM periods of lung cancer. All data represent mean ± SD, ***P<0.001 and ****P<0.0001.

**Figure 4:**
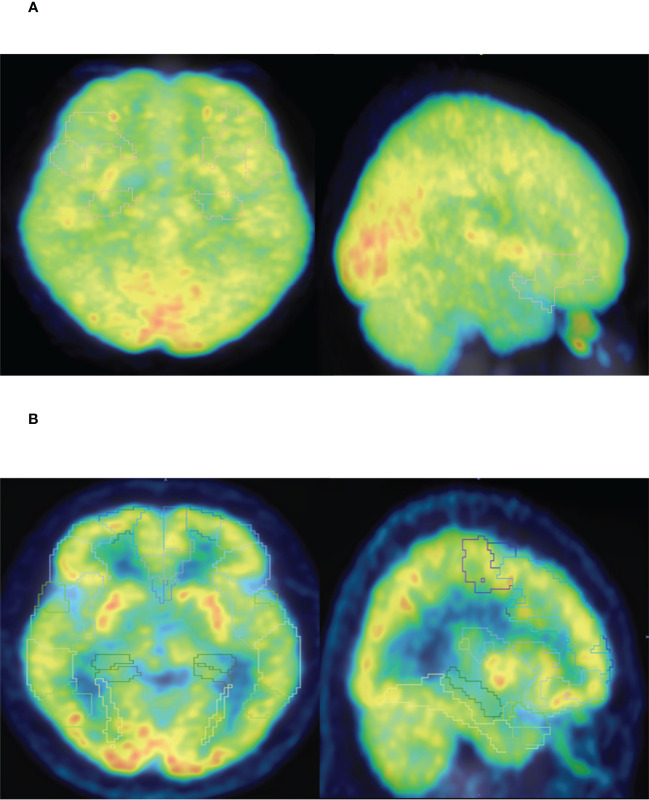
**(A)** PET-CT representative diagram of stage I patients. **(B)** PET-CT representative diagram of stage IV patients. Low metabolism is framed in different brain regions (SUV<6).

The results showed that HAMD score of patients with TNM stage IV lung cancer was significantly higher than that of patients with stage I, II and III lung cancer (**Figure 3A**). Compared to patients with TNM stage I, II and III lung cancer, MAS score of patients with stage IV tumor was significantly increased (**Figure 3B**). In addition, we identified that the SUVs and metabolic volume of bilateral frontal lobes, bilateral temporal lobes, bilateral hippocampus, bilateral caudate nucleus and left cingulate gyrus in TNM stage IV patients were significantly lower than those in stage I patients (**Figure 4**, **Tables 6**, **7**).

**Table 6:** SUV values in different TNM stages.

	I	II	P	III	P	IV	P
L-frontal	6.857 ± 0.733	6.735 ± 0.781	0.9563	6.875 ± 1.130	>0.9999	5.390 ± 1.268	<0.0001
R-frontal	7.035 ± 0.796	6.852 ± 0.811	0.8743	6.927 ± 1.201	0.9840	5.513 ± 1.226	<0.0001
L-temporal	6.962 ± 0.861	6.801 ± 1.127	0.9269	6.804 ± 1.054	0.9623	5.631 ± 1.291	<0.0001
R-temporal	7.281 ± 0.760	7.021 ± 1.078	0.7673	7.095 ± 0.955	0.9427	5.948 ± 1.402	<0.0001
L-caudate	7.740 ± 0.990	7.667 ± 1.074	0.9913	7.490 ± 1.003	0.8624	6.790 ± 1.173	0.0018
R-caudate	7.842 ± 0.845	7.851 ± 0.997	>0.9999	7.545 ± 0.773	0.7473	6.981 ± 1.133	0.0020
L-hippocampus	6.853 ± 0.664	6.655 ± 0.763	0.7308	6.968 ± 0.721	0.9607	6.010 ± 0.928	0.0002
R- hippocampus	6.989 ± 0.677	6.752 ± 0.819	0.6308	6.995 ± 0.675	0.9990	6.103 ± 0.952	0.0001
L-cingulum	8.103 ± 0.646	8.188 ± 0.518	0.9499	8.011 ± 0.536	0.9658	7.590 ± 0.780	0.0080

**Table 7:** Metabolic volume in different TNM stages.

	I	II	P	III	P	IV	P
L-frontal	12.03 ± 1.371	11.93 ± 1.696	0.9974	10.84 ± 2.344	0.2614	10.26 ± 2.038	0.001
R-frontal	12.32 ± 1.392	12.37 ± 1.481	0.9998	11.36 ± 1.891	0.5067	9.962 ± 2.474	<0.0001
L-temporal	14.75 ± 1.414	14.24 ± 1.721	0.7380	13.52 ± 1.950	0.2807	12.91 ± 1.980	0.0004
R-temporal	15.17 ± 1.361	14.83 ± 1.936	0.9065	13.88 ± 1.829	0.1841	13.23 ± 1.992	0.0002
L-caudate	9.829 ± 0.8366	9.383 ± 1.059	0.3664	8.794 ± 1.202	0.0166	8.401 ± 0.9289	<0.0001
R-caudate	10.05 ± 0.5951	9.542 ± 0.9181	0.1080	9.040 ± 0.9589	0.0026	8.686 ± 0.7902	<0.0001
L-hippocampus	9.215 ± 0.7173	8.677 ± 0.8766	0.0824	8.461 ± 0.7277	0.0409	8.045 ± 0.8089	<0.0001
R- hippocampus	9.271 ± 0.7136	8.764 ± 0.8529	0.1253	8.352 ± 0.8054	0.0095	8.189 ± 0.8480	<0.0001
L-cingulum	11.73 ± 0.9791	11.13 ± 0.6963	0.0527	10.78 ± 0.6862	0.0082	10.40 ± 0.7740	<0.0001

#### 3.3.2 Incidence rates of depression and anxiety and SUVs and metabolic volume of different brain regions in lung cancer patients with different pathological stages

**Figure 5:**
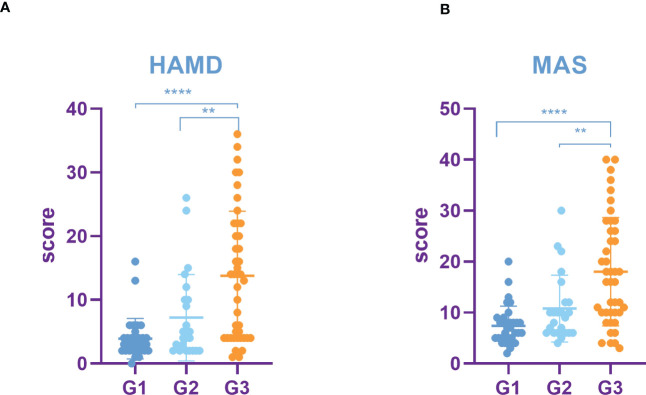
Incidence rate of depression and anxiety among lung cancer in different pathological grades. **(A)** HAMD score among different pathological grades of lung cancer. **(B)** MAS score among different pathological grades of lung cancer. All data represent mean ± SD, **P<0.01 and ****P<0.0001.

HAMD score as well as MAS score of G3 lung cancer patients was significantly higher than that of G1 and G2 (**Figures 5A, B**). Furthermore, the study showed that the SUVs and metabolic volume of bilateral frontal lobes, bilateral temporal lobes, bilateral caudate nuclei, bilateral hippocampus and left cingulate gyrus in patients with pathological grade G3 lung cancer were significantly lower than those in patients with grade G1 (**Tables 8**, **9**).

**Table 8:** SUV value between different pathological grades.

	G1	G2	P	G3	P
L-frontal	6.854 ± 0.738	6.677 ± 1.009	0.7708	5.645 ± 1.324	<0.0001
R-frontal	6.978 ± 0.820	6.848 ± 0.981	0.8691	5.754 ± 1.309	<0.0001
L-temporal	6.879 ± 0.960	6.758 ± 1.149	0.8985	5.872 ± 1.321	0.0009
R-temporal	7.229 ± 0.864	6.981 ± 1.099	0.6465	6.165 ± 1.386	0.0004
L-caudate	7.621 ± 1.036	7.696 ± 1.057	0.9545	6.933 ± 1.174	0.0180
R-caudate	7.790 ± 0.814	7.825 ± 0.986	0.9877	7.077 ± 1.124	0.0062
L-hippocampus	6.890 ± 0.671	6.589 ± 0.807	0.3069	6.183 ± 0.949	0.0010
R-hippocampus	7.000 ± 0.691	6.682 ± 0.809	0.2818	6.267 ± 0.972	0.0008
L-cingulum	8.087 ± 0.623	8.178 ± 0.533	0.8330	7.650 ± 0.783	0.0142

**Table 9:** Metabolic volume between different pathological grades.

	G1	G2	P	G3	P
L-frontal	12.05 ± 1.489	11.85 ± 1.656	0.9051	10.23 ± 2.059	0.0001
R-frontal	12.33 ± 1.422	11.65 ± 2.848	0.4431	10.44 ± 1.975	0.0005
L-temporal	14.54 ± 1.849	13.96 ± 1.933	0.4810	13.19 ± 1.769	0.0062
R-temporal	15.04 ± 1.841	14.42 ± 2.021	0.4321	13.51 ± 1.789	0.0020
L-caudate	9.549 ± 1.098	9.318 ± 1.087	0.6980	8.600 ± 1.000	0.0006
R-caudate	9.852 ± 0.8291	9.585 ± 0.7594	0.4715	8.768 ± 0.8904	<0.0001
L-hippocampus	9.072 ± 0.8646	8.712 ± 0.7767	0.2429	8.127 ± 0.8168	<0.0001
R-hippocampus	9.180 ± 0.8559	8.790 ± 0.7412	0.1823	8.182 ± 0.8164	<0.0001
L-cingulum	11.50 ± 0.9969	11.21 ± 0.8225	0.4301	10.49 ± 0.7905	<0.0001

### 3.4 Correlation analysis between HAMD, MAS score and SUVs in patients with lung cancer

**Figure 6:**
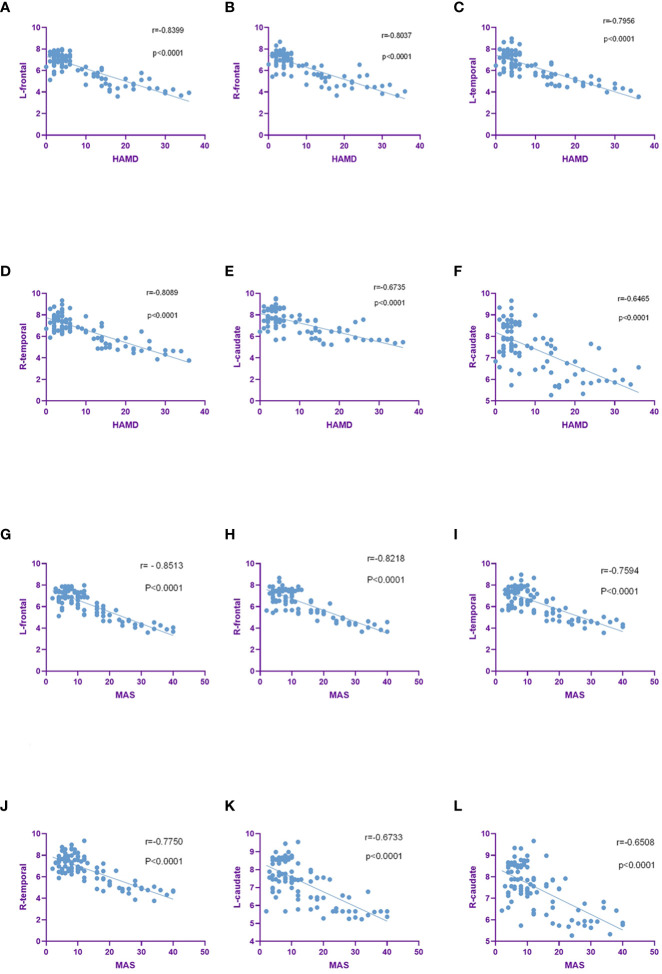
SUVs were negatively correlated with HAMD and MAS scores in the brain region. **(A–F)**. SUVs in bilateral frontal, bilateral temporal, bilateral caudate were negatively correlated with HAMD scores. **(G–L)**. SUVs in the bilateral frontal, bilateral temporal, bilateral caudate were negatively correlated with MAS scores.

We further assessed the correlation between SUVs and HAMD, MAS score. SUVs in bilateral frontal lobe, bilateral temporal lobe, bilateral caudate nucleus, bilateral hippocampus, left cingulate gyrus were negatively correlated with HAMD and MAS score. SUVs were positively correlated with HAMD and MAS score in bilateral Occipital lobe. In summary, the higher the TNM stage and pathological grade in lung cancer patients, the higher the incidence rates of depression and anxiety, and the lower the SUVs in the corresponding area (**Figures 6A–L**).

## 4 Discussion

In this study, we confirmed that the incidence of emotional disorders in lung cancer patients was higher than other four types of tumors. The incidence rate of depression and anxiety was 36.4% and 31.3% respectively and the higher TNM stage and pathological grade of lung cancer, the higher the total score of HAMD and MAS. This result was compatible with previous studies, in which lung cancer patients were commonly accompanied with depression, within a range of 11% to 44% (22). In a recent study of 104 lung cancer patients who underwent mini-interviews, 15 (14.4%) were diagnosed with depression, including major depressive disorder (MDD) (23).In our study, the incidence rates of depression and anxiety in head and neck cancer were 34.2% and 26.3% respectively, lower than those in patients with lung cancer. Depression was more prevalent in lung cancer patients than in head and neck cancer patients (24). Patients with poor pathological differentiation and advanced TNM stage had more severe disease condition, cancer-related pain and more aggressive treatment-related side effects, which put lung cancer patients at higher risks of anxiety and depression. Moreover, the duration of the tumor patient’s course also affects the severity of depression and anxiety. Moderate to severe depression and anxiety were reported in 17%and 9% of cancer survivors, respectively. One test revealed that the despair and anxiety ratings were much higher five to six years following the tumor diagnosis than they were forty weeks earlier (25). Another study revealed that there were no discernible differences between the groups with diagnoses at 5 and 10 years apart (26). Tumor itself can cause abnormal brain metabolism, resulting in emotional changes, and eventually lead to anxiety and depression. Long-term emotional, social, economic, and physical impacts on patients with chronic illnesses can also increase anxiety and depressive symptoms. In order to avoid long-term psychological and mental burden of patients, cancer patients diagnosed within one week were selected in this experiment. We found that the higher the tumor stage, the more severe the condition, and the longer the course of the disease, leading to higher HAMD and MAS scores.

This study selected patients with first-episode depression and anxiety without previous psychiatric history. In fact, depression and anxiety will recur in life. Recent meta-analyses indicated that relapse rates ranged from 18.5% to 46.5% for depression and from 13% to 42% for anxiety (27, 28). Most recurrent events occured at 6-months follow-up, with the fewest cases observed at the ninth months (29). With each new episode of recurrent depression and anxiety, the next episode tends to be more severe with more intense pessimistic and suicidal thoughts but fewer complaints of anxiety and depression (30). In addition, there are differences in imaging manifestations between first-episode depression and anxiety and recurrent depression and anxiety. A research showed that compared to first-episode depression, patients with recurrent depression had significantly reduced gray matter in the right anterior insula, right superior temporal gyrus, bilateral middle temporal gyrus, and left superior parietal gyrus (31). The comparison of regional (head and body/tail) hippocampal volumes between first episode and recurrent depressive episodes showed that the first episode had larger pretreatment hippocampal body/tail volumes (32). PET was used to detect changes in brain glucose metabolism in MDD patients, and clinical improvement in patients with first-episode depression was linked to an increase in the brain stem and cingulate and a decline in the responses of the limbic and striatal regions. The subcortical cingulate or prefrontal region of the research showed no change in recurrent depression (33). Recurrent depression and anxiety symptoms in tumor patients will lead to more complex changes in brain metabolism. We will continue to follow up the patients in this experiment to observe the changes of brain metabolism in first-episode and recurrent depression and anxiety.

In our study, an innovative graph-theoretic analysis based on FDG-PET imaging was utilized to investigate alterations of the brain metabolic in cancer patients. FDG-PET imaging can directly reflect the changes of brain glucose metabolism, and PMOD software was used to analyze abnormal metabolic brain regions in depth. In previous study, the correlation analysis between abnormal brain metabolism and the depression and anxiety showed that the frontal lobe and temporal lobe were the most important brain regions, especially frontal lobe (34, 35). Studies showed that decreased glucose uptake in the prefrontal gyrus was associated with aggression and impulsivity (36), which were prevalent in cancer patients (37). It was noted that the metabolism of the frontal lobe and temporal lobe of lung cancer patients was abnormal (38, 39). Occurrence of emotional disorders in lung cancer patients was related to the impairment of frontal temporal metabolic function (40).In this study, we discovered that SUVs and metabolic volume of patients with lung cancer and head and neck cancer were lower in bilateral frontal lobe and bilateral temporal lobe than patients with other tumors. Compared with head and neck tumors patients, brain hypometabolism was more widely involved in lung cancer patients. The metabolic decline in these brain regions was negatively correlated with the total score of HAMD and MAS. The average SUVs and metabolic volume of bilateral frontal lobe and bilateral temporal lobe were closely related to depression and anxiety. This may be part of the mechanism of the higher incidence of depression and anxiety in lung cancer patients compared with other tumors. Functional magnetic resonance imaging studies of depression also showed that (41, 42) the dysfunction of the prefrontal lobe directly affected the functional connection of the patients’ neural network. It was considered that the decrease of prefrontal lobe function may be an important neuropathological basis for the onset of depression. This study revealed that patients with lung cancer had more extensive frontal and temporal lobe damage, lower SUVs and metabolic volume, and a higher incidence of depression and anxiety. Different from previous studies, we also found other abnormal brain regions in addition to the frontal and temporal lobes, which may have great significance for further research on depression in lung cancer patients.

In addition to bilateral frontal gyrus and the bilateral temporal, decreased SUVs were observed in other cerebral regions of patients with MDD, such as the bilateral lenticular nuclei, caudate nuclei, and the bilateral anterior cingulum gyri (43). Numerous studies showed that glucose metabolism and SUVs were decreased in the caudate nucleus, putamen and anterior cingulate gyrus in patients with depression (44, 45). Similar to patients with depression, SUVs decreased in the hippocampus centrality cortex showed an alteration in the regional properties in cancer patients (46, 47). The caudate nucleus has been reported to play a pivotal role in motor processes, emotional reactivity, and executive functions in neuropsychological studies (48). Many studies on the changes in cerebral glucose metabolic rate in patients with depression supported the hypothesis that the blockage of limbic cortex pathway was related to the occurrence of depression. Qualitative changes of LCSPT were found in patients with depression, suggesting metabolic abnormality in limbic-cortex-striatum-pallidus-thalamus (LCSPT) served an important role in emotion regulation and conduction (49, 50). The prefrontal cortex, the anterior cingulum gyrus, basal ganglia, hippocampus were vital components of LCSPT (51). In this study, the SUVs decreased in hippocampus, caudate nucleus and cingulate gyrus of patients with lung cancer with anxiety and depression. Abnormal neuronal activities were noted in these cerebral regions, which were consistent with the currently recognized hypothesis of LCSPT neural circuitry. At the same time, we further manifested that the higher the TNM stage and pathological grade of lung cancer, the lower the SUVs in the corresponding areas, the more serious the neuron damage in those regions, and the higher the incidence rates and degree of anxiety and depression. It is particularly important to pay attention to the emotional changes of patients with advanced lung cancer. However, some contradictory results discovered that SUVs of patients with anxiety and depression increased in the hippocampus and anterior cingulate cortex (43, 47). These results may be due to a number of factors, such as differences in the type and stage of anxiety and depression, treatment regimens and imaging equipment. They also reflected the complexity of the etiological and neuropathological mechanisms of anxiety and depression. Due to the complexity of tumor patients and the individualization of treatment, the diagnosis and treatment of tumor depression patients were more difficult. Based on this study, early detection of abnormal brain metabolism in tumor patients may help us better identify the potential risk of depression in tumor patients. At the same time, it also provided a theoretical basis for better research on the mechanism of emotional abnormalities in cancer patients. Due to the complexity of tumor patients and the individualization of treatment, the diagnosis and treatment of tumor depression patients were more difficult. Based on this study, early detection of abnormal brain metabolism in tumor patients may help us better identify the potential risk of depression in tumor patients. At the same time, it also provided a theoretical basis for better research on the mechanism of emotional abnormalities in cancer patients.

## 5 Conclusions

This study revealed the correlation between brain glucose metabolism and emotional disorders in cancer patients, indicating that bilateral frontal temporal lobe metabolic damage may be the internal pathological basis of the occurrence of emotional disorders in cancer patients. The changes in brain glucose metabolism are expected to play a major role in the assessment of emotional disorders in cancer patients as psychobiological markers. These findings indicated that functional imaging can be applied for psychological assessment of cancer patients as an innovative method.

## Data availability statement

The original contributions presented in the study are included in the article/**Supplementary Material**. Further inquiries can be directed to the corresponding authors.

## Ethics statement

The studies involving human participants were reviewed and approved by Ethical Committee of Affiliated Hospital of Jiangnan University(LS2021090). The patients/participants provided their written informed consent to participate in this study.

## Author contributions

Conception and design of study: XY, GY. Acquisition of data: XY, YW,CY. Drafting the manuscript: XY, JW. Revising the manuscript critically for important intellectual content: RW, SZ, ZR. All authors contributed to the article and approved the submitted version.
